# Prevalence and influencing factors of anxiety and depression symptoms among the first-line medical staff in Wuhan mobile cabin hospital during the COVID-19 epidemic: A cross-sectional survey

**DOI:** 10.1097/MD.0000000000025945

**Published:** 2021-05-28

**Authors:** Xiao-Bo Zhang, Wei Xiao, Jing Lei, Ming-Xia Li, Xin Wang, Yun-Jun Hong, Ping Xu, Juan Sun

**Affiliations:** a The First People's Hospital of Changde City, Changde, Hunan Province; b Renmin Hospital of Wuhan University, Wuhan, Hubei Province, China.

**Keywords:** anxiety, COVID-19, depression, doctors, nurses

## Abstract

To investigate the prevalence of anxiety and depressive symptoms and the associated risk factors among first-line medical staff in Wuhan during the coronavirus disease 2019 (COVID-19) epidemic.

From March 5 to 15, 2020, the Hamilton Anxiety Scale and Hamilton Depression scale were used to investigate the anxiety and depression status of medical staff in Wuhan Cabin Hospital (a Hospital). Two hundred seventy-six questionnaires were received from 96 doctors and 180 nurses, including 79 males and 197 females.

During the COVID-19 epidemic, the prevalence rate of anxiety and depression was 27.9% and 18.1%, respectively, among 276 front-line medical staff in Wuhan. The prevalence rate of anxiety and depression among doctors was 19.8% and 11.5%, respectively, and the prevalence rate of anxiety and depression among nurses was 32.2% and 21.7%, respectively. Females recorded higher total scores for anxiety and depression than males, and nurses recorded higher scores for anxiety and depression than doctors.

During the COVID-19 epidemic, some first-line medical staff experienced mental health problems such as depression and anxiety. Nurses were more prone to anxiety and depression than doctors. Effective strategies toward to improving the mental health should be provided to first-line medical staff, especially female medical staff and nurses.

## 1 Introduction

Starting in December 2019, a series of patients with coronavirus disease 2019 (COVID-19) were identified in Wuhan, Hubei Province, China; this disease has become a global pandemic due to its infectivity and rapid spread across Hubei Province, the rest of China, and the world. A study published in the Lancet suggested that among 49 patients in Wuhan Jinyintan Hospital, 11% rapidly deteriorated, leading to death from multiple organ failure.^[1]^ This indirectly reflects the high death rate of COVID-19. At the moment, the therapeutic strategies to deal with the infection are only supportive, and prevention aimed at reducing transmission in the community is our best weapon.^[2]^ The World Health Organization guidelines clearly indicate that the general population is susceptible to infection with SARS-CoV-2, and the virus is easily transmitted by respiratory droplets and close contact. However, there is no vaccine to prevent infection and no specific medicine to treat the illness; only symptomatic treatments are available.^[3–5]^ In the face of this rare disease, not only the general public is alarmed, but also the medical staff were also frightened.^[6]^ According to previous studies from SARS or Ebola epidemics, the onset of a sudden and immediately life-threatening illness could lead to extraordinary amounts of pressure on healthcare workers.^[7]^ David S Hui also found that anxiety, fear, and depression caused by the high mortality rate of patients with COVID-19 and the high rate of transmission of the virus may lead to the emergence of a new and serious public health threat affecting patients, medical staff and society.^[8]^ Some investigations also have found that anxiety and depression are the most common mental health problems affecting medical staff.^[9,10]^ Facing this sudden disaster, increased workload, physical exhaustion, the risk of infection and the shortage of human resources may have dramatic effects on their physical and mental well-being. The front-line medical staff in Wuhan are, therefore, especially vulnerable to mental health problems, including anxiety, depression, and insomnia.^[11,12]^ In particular, increasing number of confirmed and suspected cases were verified in many countries outside China. Therefore, it is extremely important to realize the psychological status of the medical workforce. The purpose of our study was to investigate anxiety and depression in first-line medical personnel during the COVID-19 outbreak in Wuhan to provide an objective basis for formulating feasible psychological intervention programmes for medical staff.

## 2 Methods

### 2.1 Respondents

This study was conducted between March 5, 2020 and March 15, 2020. There were 398 first-line medical staff fighting against COVID-19 in the hospital in Wuhan. A total of 310 medical personnel volunteered to participate in the study. A questionnaire survey on personal assessment of anxiety and depression was conducted for them by specially trained medical staff face-to-face. Due to the fact that 34 medical personnel did not complete the survey (given up at midway), only 276 of the 310 questionnaires were valid. In addition, sex, age, occupation, educational background, and average monthly income were also collected. Incomplete questionnaires and participants with a history of psychological or cognitive disorder were excluded. The inclusion criteria were as follows:

(1) medical personnel who were certified to work as doctors or nurses;
(2) medical personnel engaged in the clinical diagnosis and treatment of patients with COVID-19;
(3) without a chronic disease affecting anxiety or depression; and
(4) voluntary participation in the survey.

Ethical approval was issued by the Ethics Committee of The First People's Hospital of Changde City and Wuhan mobile cabin hospital, and all the participants had signed an informed consent before the study was initiated.

### 2.2 Research tools

By using the self-designed questionnaire, we have obtained the general demographic information of the respondents, including sex, age, occupation, educational background, and average monthly income.

### 2.3 Questionnaire measurement of anxiety and depression

Hamilton Anxiety Scale (HAM-A) and Hamilton Depression scale (HAM-D) have been widely used to assess the appearance of anxiety and depression.^[13,14]^ HAM-A contains 14 questions and HAM-D contains 17 questions. Some items are scored on a 5-point scale ranging from 0 to 4 points. Each question includes 5 items. Responses are scored as 0 (never), 1 (mild), 2 (moderate), 3 (severe), or 4 (extremely serious). Overall, the total HAM-A score is operationally categorized as follows: no anxiety (score of 0–6 points), mild and moderate anxiety (score of 7–13 points), and severe anxiety (score ≥14 points). The total HAM-D score is classified into normal (score of 0–6 points), mild and moderate (score of 7–23 points), and severe depression (score ≥24 points). In addition, a higher score represents more severe anxiety symptoms. According to a previous study, these questionnaires assess the respondent's psychological condition with satisfactory reliability and validity.^[15]^

### 2.4 Statistical analyses

SPSS 11.0 statistical software was used for the data analysis. The continuous variables are presented as means ± standard deviations. HAM-A score greater than 7 points is anxiety. HAM-D score greater than 7 points is depression. Counts are described by a frequency distribution, and independent-sample *t* tests were used for comparisons between 2 groups. The factors influencing anxiety or depression were analyzed with a univariate model. Statistical significance was defined as *P* < .05 for all tests.

## 3 Results

### 3.1 Participants’ characteristics (Tables 1 and 2)

A total of 310 questionnaires were collected, 276 of which were valid. Among the 276 valid questionnaires, 79 were male and 197 were female. Their ages ranged from 18 to 65 years of age; 96 were doctors and 180 were nurses. There were 38 participants who had received a secondary specialized degree; 147 had received an undergraduate degree; 91 had received a master's degree or above. Regarding the monthly family income, 173 earned 10,000 yuan or less, 95 earned 10,000 yuan to 20,000 yuan, and 64 earned 20,001 yuan or more. The data are all available in this article.

**Table 1:** Anxiety status and sociodemographic profile of medical staff.

	N	No anxiety	Anxiety	Percentage (%)	*P*
Gender					
Male	79	60	19	24.0%(19/79)	.36
Female	197	139	58	29.4%(58/197)	
Age (yr)					
<30	112	77	35	31.2%(35/112)	
30–45	137	98	89	28.4%(39/137)	.109
>45	27	24	3	11.1%(3/27)	
Occupation					
Doctor	96	77	19	19.8%(19/96)	.028
Nurse	180	122	58	32.2%(58/180)	
Education					
Secondary specialize	38	25	13	34.2%(13/38)	
College and undergraduate	147	108	39	26.5%(39/147)	.638
Master or above	91	66	25	27.5%(25/91)	
Income (yuan)					
5000–10,000	173	120	53	30.6%(53/173)	.328
10,000–20,000	95	72	23	24.2%(23/95)	
>20,000	8	7	1	12.5%(1/8)	

**Table 2:** Depression status and sociodemographic profile of medical staff.

	N	No anxiety	Anxiety	Percentage (%)	*P*
Gender					
Male	79	65	14	17.7% (14/79)	.91
Female	197	161	36	18.3.%(36/197)	
Age (yr)					
<30	112	92	20	17.9%(20/112)	
30–45	137	112	25	18.2%(25/137)	.995
>45	27	22	5	18.5%(5/27)	
Occupation					
Doctor	96	85	11	11.5%(11/96)	.036
Nurse	180	141	39	21.7%(39/180)	
Education					
Secondary specialize	38	30	8	21.1%(8/38)	
College and undergraduate	147	120	27	20.9%(27/147)	.823
Master or above	91	76	15	14.8%(15/91)	
Income (yuan)					
5000–10,000	173	139	34	19.79%(34/173)	.673
10,000–20,000	95	80	15	15.8%(15/95)	
>20,000	8	7	1	12.5%(1/8)	

### 3.2 Anxiety and depression in front-line medical staff (Tables 1 and 2)

Anxiety was identified in 77 of the 276 medical staff, with a prevalence rate of 27.9%. The prevalence rate of anxiety in nurses was 32.2% and that in doctors was 19.8%. Depression was identified in 50 of the 276 medical staff, with the prevalence rate of 18.1%. The prevalence rate of depression was 21.7% in nurses and 11.5% in doctors.

### 3.3 Analysis of anxiety and depression scores in medical staff of different sexes and occupations (Table 3)

Among the 276 medical staff who responded, the anxiety scores in males and females were 6.22 ± 1.61 and 6.82 ± 2.17, respectively. The depression scores in males and females were 6.57 ± 1.44 and 7.39 ± 2.13, respectively. The anxiety score in doctors was 5.75 ± 1.72 and that in nurses was 6.41 ± 2.14. The depression score in doctors was 6.79 ± 1.38 and that in nurses was 7.62 ± 1.82. The scores for anxiety and depression were higher in females than in males and higher in nurses than in doctors, and the differences were statistically significant (*P* < .05).

**Table 3:** Analysis of anxiety and depression scores of different genders and occupational medical staff.

	Gender		*P*	Occupation		*P*
	Male	Female		Doctor	Nurse	
HAM-A scores (mean ± SD)	6.22 ± 1.61	6.82 ± 2.17	.027	5.75 ± 1.72	6.41 ± 2.14	.009
HAMD scores (mean ± SD)	6.57 ± 1 44	7.39 ± 2.13	.001	6.79 ± 1.38	7.62 ± 1.82	.001

### 3.4 Association between potential risk factors and anxiety and depression (Tables 4 and 5)

Univariate analysis described the odds ratio (OR) of potential related factors and the corresponding 95%CI. The results showed that compared with doctors, the prevalence rate of anxiety of professional nurses increased by 93% (OR = 1.93, *P* = .0298, 95%CI: 1.07, 3.48). Women seem to be more prone to anxiety, but there was no statistical significance. However, age, education, and income were not found to be correlated with anxiety. The risk of depression in nurses was 2.14 times that of doctors (OR = 2.14, *P* = .039, 95%CI: 1.04, 4.4). No correlation was found between age, education, or income and depression.

**Table 4:** Association between potential risk factors and anxiety.

Variable	N (%)	OR (95%CI)	*P*
Gender			
Male	79 (28.62%)	1	
Female	197 (71.38%)	1.45 (0.79, 2.66)	.2318
Age (yr)			
<30	112 (40.58%)	1	
30–45	137 (49.64%)	0.95 (0.55, 1.63)	.8421
>45	27 (9.78%)	0.29 (0.08, 1.02)	.0531
Occupation			
Doctor	96 (34.78%)	1	
Nurse	180 (65.22%)	1.93 (1.07, 3.48)	.0298
Education			
Secondary specialize	38 (13.77%)	1	
College and undergraduate	147 (53.26%)	0.69 (0.32, 1.49)	.3494
Master or above	91 (32.97%)	0.73 (0.32, 1.64)	.445
Income (yuan)			
5000–10,000	173 (62.68%)	1	
10,000–20,000	95 (34.42%)	0.72 (0.41, 1.28)	.2653
>20,000	8 (2.90%)	0.32 (0.04, 2.70)	.2967

**Table 5:** Association between potential risk factors and depression.

Variable	N (%)	OR (95%CI)	*P*
Gender			
Male	112 (40.58%)	1	
Female	137 (49.64%)	1.03 (0.54, 1.97)	.9364
Age (yr)			
<30	27 (9.78%)	1.05 (0.35, 3.09)	.936
30–45	79 (28.62%)	1	
>45	197 (71.38%)	1.04 (0.53, 2.05)	.9142
Occupation			
Doctor	96 (34.78%)	1	
Nurse	180 (65.22%)	2.14 (1.04, 4.40)	.039
Education			
Secondary specialize	38 (13.77%)	1	
College and undergraduate	147 (53.26%)	0.84 (0.35, 2.04)	.7066
Master or above	91 (32.97%)	0.74 (0.28, 1.93)	.5375
Income (yuan)			
5000–10,000	173 (62.68%)	1	
10,000–20,000	95 (34.42%)	0.77 (0.39, 1.49)	.4346
>20,000	8 (2.90%)	0.58 (0.07, 4.91)	.6205

## 4 Discussion

In December 2019, COVID-19 began in Wuhan (Hubei, China) and attracted worldwide attention.^[16]^ Soon afterwards, the WHO has classified COVID-19 as a major disaster and global pandemic. As shown in a previous study,^[17]^ disasters create some degree of mental health problems. Recent data from a public opinion survey show that COVID-19 has exerted significant effects on mental health.^[18]^ When confronted with a new infectious disease, the reasons for the psychological distress to which medical health workers are exposed might be related to the many difficulties of remaining safe at work, such as the initially insufficient understanding of the virus, the lack of prevention and control knowledge, the long-term workload, the high risk of exposure to patients with COVID-19, and the shortage of medical protective equipment.^[19,20]^ Thus, the occupational exposure risk is very high for front-line medical staff.^[21,22]^ During the fight against COVID-19, front-line medical personnel are unable to be reunited with their family and may feel afraid, lonely, anxious, and depressed. They must be isolated in a hotel after work, and thus their normal life is restricted. Caregivers for patients with novel infectious diseases have little knowledge of the spread of the disease, effective treatment options for the disease, best practices to care for these patients, and methods to adapt to the pressures imposed by the epidemic.^[6,23,24]^ These specific situations impose a considerable amount of stress on the medical staff, which might lead to high levels of psychological distress. As shown in the present study, the prevalence of anxiety and depression in medical staff was 27.9% and 18.1%, respectively. The prevalence of anxiety and depression in doctors was 19.8% and 11.5%, respectively, and the prevalence of anxiety and depression in nurses was 32.2% and 21.7%, respectively. Clearly, the prevalence of anxiety and depression was higher in nurses than in doctors (OR = 1.93, *P* = .0298, 95%CI: 1.07, 3.48) and (OR = 2.14, *P* = .039, 95%CI: 1.04, 4.4). Therefore, the nursing occupation is an important risk factor for anxiety and depression. The prevalence of anxiety in this study was similar to the results of the study by Huang.^[23]^ The prevalence of anxiety was higher in nurses than in doctors; in contrast, the prevalence of depression was lower in nurses than in doctors.^[9]^ However, these results were different from the prevalence of anxiety and depression reported in first-line medical staff in Australia in 2018,^[25]^ and this difference might be attributed to the use of different investigation tools and different sample sizes. However, age, education, and income were not correlated with anxiety and depression, consistent with the results of the study by Huang.^[23]^ Medical staff in Wuhan had different degrees of stress and anxiety related to COVID-19, and the anxiety level was significantly correlated with the stress level, which exerted negative effects on self-efficacy and sleep quality.^[26]^ Based on the results of the present study comparing the average values of anxiety and depression between doctors and nurses, nurses presented more anxiety and depression than doctors. The potential explanation is that nurses have more responsibilities and work longer in isolation wards than doctors; in addition, nurses have more contact with patients, more infection opportunities, and a heavier ideological burden and are more prone to fatigue and tension.^[27]^

In addition, the prevalence of anxiety and depression in males was 24% and 17.7%, respectively. The prevalence of anxiety and depression in females was 29.4% and 18.3%, respectively. Although the differences were not statistically significant, females were more prone to anxiety. The anxiety scores of males and females were 6.22 ± 1.61 and 6.82 ± 2.17, respectively, and the depression scores of males and females were 6.57 ± 1.44 and 7.39 ± 2.13, respectively. However, the anxiety and depression scores of females were higher than males, indicating that females experienced more severe anxiety and depression. A considerable number of surveys have indicated that depression is more obvious in females, which may be related to the fact that females pay more attention to their inner feelings and their psychological and social states.^[23,28]^ The high scores on the anxiety and depression scales were significantly correlated with the heavy workload experienced by females, the number of hours spent working each week, and the number of night shifts per month in a previous study.^[29]^ No significant differences in anxiety and depression scores were observed among medical staff of different ages, income levels, and educational backgrounds, consistent with the results of the study by Huang.^[23]^

We must admit that our study has certain limitations. First, the sample size analyzed is relatively small, which reduces the reliability of the study. Second, all medical staff were employed from a hospital, and thus the results might not be generalized to all medical staff in China and even in the world. Future research will still need to potentially include longitudinal tracking of the factors and an evaluation of the effects of therapeutic interventions.

## 5 Conclusions

In conclusion, the current study described the anxiety and depression symptoms occurring in the frontline medical staff in Wuhan during the COVID-19 epidemic. Nurses were more prone to anxiety and depression than doctors, and female medical staff may experience significantly more severe anxiety and depression than male medical staff. Timely psychological interventions should be implemented in the early stages to avoid greater psychological harm to medical staff, which might lead to adverse emotions and affect the quality of their medical work. For the future advancement of a hospital in China, we should advocate a people-oriented culture and closely monitor the mental health of medical staff, particularly female medical staff and nurses. Based on their psychological problems, an institution providing comprehensive psychological consultations should be established, and the mental health management of medical staff should continue for an extended period.^[15]^

## Acknowledgments

The authors wish to thank medical staff for their contributions to the study.

## Author contributions

**Conceptualization:** Xiao-Bo Zhang, Jing Lei.

**Data curation:** Jing Lei, Ming-Xia Li, Juan Sun.

**Formal analysis:** Xiao-Bo Zhang, Xin Wang, Yun-Jun Hong, Ping Xu.

**Funding acquisition:** Xiao-Bo Zhang.

**Investigation:** Wei Xiao, Jing Lei, Ming-Xia Li, Juan Sun.

**Project administration:** Jing Lei, Ming-Xia Li.

**Supervision:** Xiao-Bo Zhang, Wei Xiao.

**Writing – original draft preparation:** Xiao-Bo Zhang.

**Writing – review & editing:** Xiao-Bo Zhang, Wei Xiao, Xin Wang, Ping Xu.
